# Real‐Time Ventricular Fibrillation Amplitude‐Spectral Area Analysis to Guide Timing of Shock Delivery Improves Defibrillation Efficacy During Cardiopulmonary Resuscitation in Swine

**DOI:** 10.1161/JAHA.117.006749

**Published:** 2017-11-04

**Authors:** Salvatore Aiello, Michelle Perez, Chad Cogan, Alvin Baetiong, Steven A. Miller, Jeejabai Radhakrishnan, Christopher L. Kaufman, Raúl J. Gazmuri

**Affiliations:** ^1^ Rosalind Franklin University of Medicine and Science North Chicago IL; ^2^ Resuscitation Institute Rosalind Franklin University of Medicine and Science North Chicago IL; ^3^ Scientific Affairs at ZOLL Medical Corporation Chelmsford MA; ^4^ Critical Care Medicine Captain James A. Lovell Federal Health Care Center North Chicago IL

**Keywords:** amplitude spectral area, animal model, defibrillation, resuscitation, sudden cardiac arrest, ventricular fibrillation, ventricular fibrillation waveform analysis, waveform analysis, Cardiopulmonary Resuscitation and Emergency Cardiac Care, Sudden Cardiac Death, Ventricular Fibrillation, Animal Models of Human Disease, Translational Studies

## Abstract

**Background:**

The ventricular fibrillation amplitude spectral area (AMSA) predicts whether an electrical shock could terminate ventricular fibrillation and prompt return of spontaneous circulation. We hypothesized that AMSA can guide more precise timing for effective shock delivery during cardiopulmonary resuscitation.

**Methods and Results:**

Three shock delivery protocols were compared in 12 pigs each after electrically induced ventricular fibrillation, with the duration of untreated ventricular fibrillation evenly stratified into 6, 9, and 12 minutes: AMSA‐Driven (AD), guided by an AMSA algorithm; Guidelines‐Driven (GD), according to cardiopulmonary resuscitation guidelines; and Guidelines‐Driven/AMSA‐Enabled (GDAE), as per GD but allowing earlier shocks upon exceeding an AMSA threshold. Shocks delivered using the AD, GD, and GDAE protocols were 21, 40, and 62, with GDAE delivering only 2 AMSA‐enabled shocks. The corresponding 240‐minute survival was 8/12, 6/12, and 2/12 (log‐rank test, *P*=0.035) with AD exceeding GDAE (*P*=0.026). The time to first shock (seconds) was (median [Q1–Q3]) 272 (161–356), 124 (124–125), and 125 (124–125) (*P*<0.001) with AD exceeding GD and GDAE (*P*<0.05); the average coronary perfusion pressure before first shock (mm Hg) was 16 (9–30), 10 (6–12), and 3 (−1 to 9) (*P*=0.002) with AD exceeding GDAE (*P*<0.05); and AMSA preceding the first shock (mV·Hz, mean±SD) was 13.3±2.2, 9.0±1.6, and 8.6±2.0 (*P*<0.001) with AD exceeding GD and GDAE (*P*<0.001). The AD protocol delivered fewer unsuccessful shocks (ie, less shock burden) yielding less postresuscitation myocardial dysfunction and higher 240‐minute survival.

**Conclusions:**

The AD protocol improved the time precision for shock delivery, resulting in less shock burden and less postresuscitation myocardial dysfunction, potentially improving survival compared with time‐fixed, guidelines‐driven, shock delivery protocols.

**Clinical Perspective**

**What Is New?**

- Currently, the decision to deliver an electrical shock during resuscitation from ventricular fibrillation is binary based on the presence or absence of a “shockable” rhythm.
- We developed an alternative approach using real‐time analysis of the ventricular fibrillation waveform amplitude spectral area to guide when to deliver a shock based on the probability that the shock could terminate ventricular fibrillation and reestablish spontaneous circulation.
- The amplitude spectral area–driven approach, compared with guidelines‐driven protocols, was more effective and reduced the shock burden (ie, cumulative number of ineffective shocks) resulting in less postresuscitation myocardial dysfunction and better short‐term survival.

**What Are the Clinical Implications?**

- The study provides support for translational studies in sudden cardiac arrest investigating whether amplitude spectral area–driven defibrillation protocols—by timing the defibrillation effort to myocardial readiness for successful defibrillation—could be more effective than time‐fixed guidelines‐driven protocols resulting in less shock burden, less postresuscitation myocardial dysfunction, and better survival.
- The study also supports investigating amplitude spectral area for guiding hemodynamic optimization of the resuscitation effort.

Current guidelines for resuscitation from ventricular fibrillation (VF) recommend delivery of an electrical shock as soon as a defibrillator is available and every 2 minutes thereafter if VF persists.^1^ This approach treats defibrillation as a binary choice between “shockable” and “nonshockable” rhythms. However, not all shocks are successful in terminating VF and securing the return of spontaneous circulation (ROSC). Preclinical and clinical studies have shown that duration of untreated VF^2^, ^3^, ^4^, ^5^ and the level of coronary blood flow generated by cardiopulmonary resuscitation (CPR) are primary determinants of shock success.^6^, ^7^, ^8^, ^9^, ^10^ Thus, shocks delivered early after prolonged untreated VF and/or during hemodynamically ineffective CPR are not likely to be effective^7^, ^11^ and may instead be detrimental by prompting interruptions in chest compression and causing electrical injury to the myocardium despite the widespread adoption of biphasic waveform defibrillation.^12^, ^13^ Accordingly, it would be desirable if the decision to deliver electrical shocks were not binary—based on the presence or absence of a “shockable” rhythm—but instead relied on the probability that a shock could result in the termination of VF followed by ROSC.

Analysis of the VF waveform in the frequency domain calculating its amplitude spectral area (AMSA) has been shown to inform on the likelihood that ROSC may occur after delivery of an electrical shock.^9^, ^14^, ^15^, ^16^, ^17^, ^18^, ^19^, ^20^ AMSA reflects, in part, the energy state of the myocardium^21^, ^22^ and its readiness for successful defibrillation.^20^, ^23^, ^24^ The highest value of AMSA is typically observed at the onset of VF. It declines during untreated VF and increases again during CPR, as the myocardium is being reperfused, reaching a plateau after ≈6 minutes of CPR.^25^, ^26^, ^27^, ^28^

We therefore hypothesized that real‐time monitoring of AMSA during ventilation pauses of CPR could more effectively guide the decision about when to deliver an electrical shock and thereby improve resuscitation outcomes. We tested this hypothesis in a swine model of electrically induced VF and resuscitation by conventional CPR using a basic life support protocol and compared 3 defibrillation protocols: (1) an AMSA‐Driven (AD) protocol, (2) a Guidelines‐Driven (GD) protocol, and (3) a Guidelines‐Driven/AMSA‐Enabled (GDAE) protocol that allowed earlier shock delivery upon exceeding an AMSA threshold.

## Methods

The study was approved by the Institutional Animal Care and Use Committee at Rosalind Franklin University of Medicine and Science (protocol #16‐06) and was conducted according to institutional guidelines.

### Study Design

Three groups of 12 pigs each (total, n=36 pigs) were block‐randomized to 1 of 3 defibrillation protocols. The duration of untreated VF was stratified into 6, 9, and 12 minutes to model variable downtime yielding 9 unique combinations of untreated VF durations and defibrillation protocols per block. The assignment was revealed only after completion of the surgical preparation and before induction of VF.

#### Defibrillation protocols

**Figure 1:**
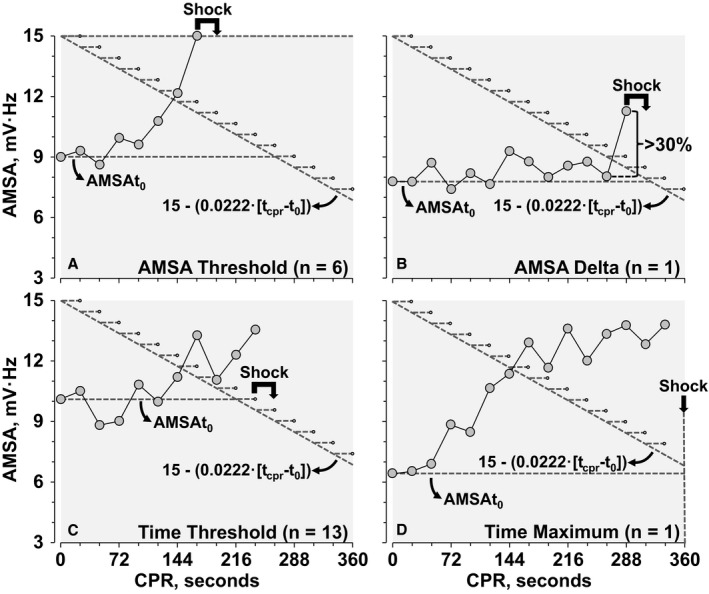
AMSA‐Driven (AD) protocol featuring 1 representative experiment for each shock advisory criterion and the corresponding total number of shocks given guided by such criterion for the entire AD group. Shown in each graph are the AMSA at the time CPR started (AMSAt_0_) and the time threshold according to: 15−(0.0222·[t_cpr_−t_0_]); where t_0_ is the time at which CPR started and t_cpr_ is the CPR time in seconds with each “step” representing the interval between ventilation pauses during which shock advise and subsequent shock delivery occurred (if advised). The AMSA threshold criterion (A) and AMSA delta criterion (B) are time independent based on reaching an AMSA value ≥15 mV·Hz (AMSA threshold) or having an AMSA increase ≥30% relative to the preceding AMSA value at any time during CPR (AMSA delta). The time threshold criterion (C) and the time maximum criterion (D) are independent of the AMSA level during CPR but dependent on CPR duration, with the time threshold setting a time to advise shock based on AMSAt_0_ exceeding 15−(0.0222·[t_cpr_−t_0_] in seconds) and the time maximum setting a time (360 s) by which a shock must be delivered. As shown by the representative experiments, the threshold criterion (A) and the delta criterion (B) were met before the time threshold criterion. The time threshold criterion was met after failing to meet AMSA threshold or AMSA delta criterion while having an AMSAt_0_ that enabled shock advice within 360 s (C). When none of the above criteria were met, the time maximum criterion advised shock at 360 s (D). Please refer to the Methods section for the approach developed to reset the algorithm after a shock is advised. AMSA indicates amplitude spectral area; CPR, cardiopulmonary resuscitation.

With the AD protocol, shocks were advised according to a 4‐criterion algorithm. Two criteria were time independent and advised shock delivery when AMSA equaled or exceeded 15 mV·Hz (*AMSA threshold*) or when an increase in AMSA equaled or exceed by 30% the preceding AMSA value (*AMSA delta*). The 2 remaining criteria were time dependent. The first time‐dependent criterion advised shock delivery when a CPR time (t_cpr_)—in seconds—was exceeded (*time threshold*). t_cpr_ was defined as the time during CPR when the AMSA measured immediately before starting CPR (AMSA at time zero, AMSAt_0_) exceeded 15−(0.0222·[t_cpr_−t_0_] in seconds). The second time‐dependent criterion established a maximum of 360 s from AMSAt_0_ for a shock to be advised if no other advisory criteria had been met (*time maximum*). If a shock was advised, the shock was delivered at the subsequent pause (ie, ≈18 s later), also allowing for charging of the defibrillator without interrupting chest compressions (Figure 1). The shock advisory algorithm was reset starting at the pause following the delivered shock. For the *time threshold* criterion, “0.0222·[t_cpr_−t_0_]” was modified to “0.0222·[t_cpr_−t_pshock‐AMSA_],” where t_pshock‐AMSA_ indicates the time at which the first postshock AMSA was measured. For the *time maximum* criterion, “AMSAt_0_” was modified to “t_pshock‐AMSA_.”

With the GD protocol, shocks were delivered guided by the 2015 resuscitation guidelines.^29^, ^30^ Thus, shocks were delivered every 2 minutes during a ventilation pause if VF was present for a maximum of 8 shocks given the corresponding maximum 16‐minute duration of CPR. With the GDAE protocol, shocks were delivered as per GD protocol but allowing earlier shock delivery if AMSA equaled or exceeded 15 mV·Hz. The shock was aborted if immediately before shock delivery VF was no longer present.

### Software Applications

Custom applications were developed using LabVIEW system design software (Version 6.0, National Instruments, Austin, TX) that allowed sampling and digitizing transduced signals at 250 Hz using a 16‐channel, 16‐bit data acquisition board with 8 digital I/O lines (AT‐MIO‐16XE‐50; National Instruments). The application allowed simultaneous monitoring and recording signals, calculating AMSA, advising when to deliver a shock, and controlling the mechanical compressor using digital I/O lines programmed to activate (5 V) and deactivate (0 V) a push solenoid electromagnet (Model 10907; Uxcell, Hong Kong, China) actuating on the compressor on/off switch.

To calculate AMSA, the VF signal was analyzed in the frequency domain after fast Fourier transform applying a Tukey window to reduce edge effects. AMSA was determined as the summed product of individual frequencies (F_i_) and their corresponding amplitudes (A_i_) within a 2‐ to 48‐Hz window multiplied by a scaling factor; ie, AMSA=1.704·Σ(A_i_·F_i_), reported in mV·Hz.

The AMSA value used for this application involved (1) calculation of AMSA from a 2.1‐s VF segment (ie, 525 data points at the sampling rate of 250 Hz), (2) performing 475 sequential iterations of the AMSA calculations over a 4‐s interval (ie, 1000–525 data points) shifting 1 data point forward after each iteration, and (3) storing the lowest of the 475 AMSA calculations to be used by the shock advisory algorithm described above under Study Design. To avoid or minimize compression artifacts, the signal used for AMSA calculations was acquired during the last 4 s of each ventilation pause.

### Animal Preparation

**Figure 2:**
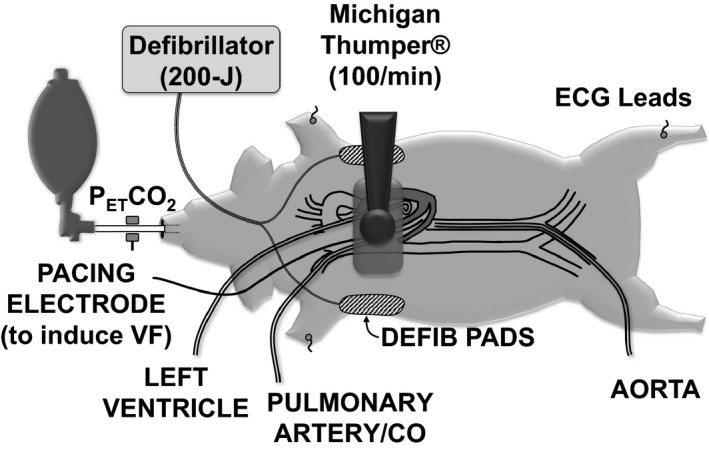
Swine model of electrically induced VF with resuscitation attempted by conventional CPR using a mechanical piston device and bag‐valve ventilation through an endotracheal tube with delivery of 100% oxygen. CPR indicates cardiopulmonary resuscitation; VF, ventricular fibrillation.

Male domestic pigs (35.3–44.5 kg) were sedated with ketamine hydrochloride (30 mg/kg, intramuscular) and anesthesia induced with propofol (2 mg/kg, through an ear vein). The trachea was intubated with a size 7.5 orotracheal tube and positive pressure ventilation initiated with 50% oxygen using a volume‐controlled ventilator (840 Ventilator System; Nellcor Puritan Bennett, Boulder, CO) set to deliver a tidal volume of 10 mL/kg (peak flow 60 L/min). The respiratory rate was adjusted at baseline to attain an end‐expired pco_2_ (P_E_ _t_co_2_) between 38 and 42 mm Hg (Capnogard; Novametrix Medical Systems, Wallingford, CT). Anesthesia was maintained with isoflurane (1.0–3.5%). ECG signals were obtained through pads from a biphasic waveform defibrillator (E series; ZOLL Medical Corporation, Chelmsford, MA) placed on the shaved sides of the chest along the midclavicular line and through leads placed on the right forelimb, left forelimb, and left hindlimb. The ECG from the limb leads was input and recorded during spontaneous circulation, whereas the ECG from the chest pads was input and recorded for AMSA analysis. For hemodynamic monitoring and blood sampling, a 6F fluid‐filled catheter (Langston 5550; Vascular Solutions, Minneapolis, MN) was advanced from the right femoral artery into the descending thoracic aorta; a 7F thermodilution balloon‐tipped pulmonary artery catheter (Edwards Critical Care Explorer; Baxter Healthcare Corp) was advanced through the right external jugular into the pulmonary artery, and a 6F fluid‐filled pigtail catheter (Langston 5540; Vascular Solutions) was advanced from the right carotid artery into the left ventricle. A 5F pacing electrode (008556P; Bard Medical, Covington, GA) was advanced through the right internal jugular vein into the right ventricle and used for induction of VF (Figure 2). Core temperature was measured through the pulmonary artery catheter and was maintained between 37.5 and 38.5°C using a servo‐controlled water‐circulated blanket (Blanketrol II; Cincinnati Sub‐Zero, Cincinnati, OH).

### Resuscitation Protocol

VF was induced by delivering an alternating current (1–10 mA) to the right ventricular endocardium and left untreated without ventilations for a predetermined interval as described in Study Design. At the end of untreated VF, chest compressions were started using a mechanical compressor (Thumper Model 1007; Michigan Instruments, Grand Rapids, MI) factory‐programmed to deliver 100 compressions per minute. To simulate the basic life support protocol, we retrofitted the chest compressor with a computer‐controlled device that actuated on its on‐off switch (as described under Software Applications) pausing every 18 s (ie, after delivering 30 compressions) for 6 s to manually deliver 2 ventilations with 100% oxygen using a 350‐mL self‐inflating pediatric bag (Lifesaver Resuscitation Bags; Teleflex Medical, NC). Chest compressions were adjusted within the initial 120 s, attempting to generate an aortic diastolic pressure of at least 25 mm Hg by first increasing the force of compression up to a depth of 5 cm and subsequently—if needed—by repositioning the animal laterally and/or rostrocaudally relative to the piston during ventilation pauses. Rectilinear biphasic 200‐J electrical shocks were delivered at fixed‐dose through the chest pads during ventilation pauses according to 1 of 3 protocols described under Study Design. Ventilations were held during shock delivery. Chest compressions were resumed if VF persisted or the mean aortic pressure was <25 mm Hg immediately after the shock. If an electrically organized rhythm was recognized with a mean aortic pressure ≥25 mm Hg, chest compressions were held to determine whether ROSC had occurred; otherwise, chest compressions were resumed. If ROSC had not been achieved after 4 minutes of CPR, epinephrine—1 mg‐bolus—was delivered into the right atrium followed by a 10‐mL 0.9% NaCl flush. The epinephrine dose was repeated at 8 and 12 minutes if ROSC had not been achieved and VF persisted. The resuscitation effort was stopped after VF was terminated or after 16 minutes of CPR. VF that recurred after ROSC was treated by delivering up to 10 electrical shocks without additional chest compression. Animals that achieved ROSC received 0.9% NaCl (30 mL/kg) at the maximum pump rate (999 mL/h) and were monitored for up to 240 minutes, euthanizing those that survived by delivering a right atrial bolus of KCl (150 mg/kg).

### Measurements

Pressures were measured through fluid‐filled systems. Cardiac output was measured in duplicate after 10‐mL bolus injection of 0.9% NaCl into the right atrium (Edwards Lifesciences Vigilance model, Irvine, CA) and normalized to body surface area using the Kelley equation (body surface area [m^2^]=0.073·body‐weight^2/3^ [kg]).^31^ The coronary perfusion pressure during CPR was calculated as the difference between aortic and right atrial pressure at the end of the interval between compressions. Blood samples collected from the aorta and pulmonary artery were processed on site for pH, po_2_, pco_2_, hemoglobin, base excess, and lactate using a cartridge‐based device (OPTI CCA‐TS Blood Gas and Electrolyte Analyzer; OPTI Medical Systems, Roswell, GA) and for common hemoglobin types (oxy‐, met‐, carboxy‐, and reduced‐) using a co‐oximeter (AVOXimeter 4000; A‐VOX Systems Inc, San Antonio, TX). Pertinent indices of cardiovascular and metabolic function were derived from these primary measurements as previously described.^32^, ^33^, ^34^

### Outcomes

The immediate outcome after each shock was determined at the subsequent ventilation pause or equivalent time if compressions were not resumed (ie, ≈18 s after the shock) and classified as (1) VF_*p*_, persistency of VF; (2) ROSC, organized pulsatile cardiac rhythm with a mean aortic pressure ≥40 mm Hg; (3) return of cardiac activity, organized pulsatile cardiac rhythm with a mean aortic pressure ≥25 <40 mm Hg; and (4) pulseless electrical activity, organized pulsatile or nonpulsatile cardiac rhythm with a mean aortic pressure <25 mm Hg. Shock burden was defined as the cumulative energy of shocks delivered that failed to achieve immediate ROSC according to: Shock burden (J/kg)=J/Weight (kg)·(no‐ROSC shocks[n]); where no‐ROSC denotes the number of shocks that resulted in either return of cardiac activity, pulseless electrical activity, or persistent VF (VF_*p*_). Successful resuscitation was defined as achieving ROSC lasting >5 minutes; and survival as remaining hemodynamically viable at 240 minutes postresuscitation.

### Statistical Analysis

Continuous time‐invariant dependent variables were analyzed by 1‐way ANOVA using the Holm–Sidak test for pairwise comparisons if the data were normally distributed or by the Kruskal–Wallis test on Ranks using the Tukey test for pairwise comparisons if the data were not normally distributed (ie, failing the Shapiro–Wilk and/or the Equal Variance test), correspondingly reporting means±SD or median with the interquartile range. Proportions were analyzed by Pearson's χ^2^ test. Kaplan–Meier survival curves were analyzed by log‐rank test using the Holm–Sidak test for pairwise comparisons and survival time was analyzed by the Cox regression method. The effect of the interaction between duration of untreated VF and defibrillation protocol on survival time was analyzed by 2‐way ANOVA. Linear regression was used to analyze the relationship between shock burden and both post‐ROSC survival time and left ventricular stroke work index (SigmaPlot 12.5; Systat Software, Inc).

Receiver operator characteristic curves were used to analyze the relationship between preshock AMSA and postshock ROSC. Continuous time‐varying dependent variables were analyzed using linear mixed‐effect models treating time as a continuous variable to assess main effects and interactions. Comparisons at each time point were obtained by treating each time point as a discrete moment mainly for descriptive reasons. The Sidak pairwise multiple comparisons method was applied when appropriate. Linear mixed‐effect models are currently preferred for studies with repeated measurements as mixed‐effect models handle missing data (without listwide deletion).^35^, ^36^ Restricted maximum likelihood estimation was used to fit a linear mixed‐effect model and examine variables that could have predicted each of the AMSA values measured during CPR with the goal of identifying time‐invariant and time‐variant predictors. To identify time‐invariant predictors, we included variables shown in Table to differ at baseline among defibrillation protocols (ie, weight, arterial pH, and arterial pco_2_), the duration of untreated VF, and the pre‐CPR AMSA. To identify time‐variant predictors, we included variables expected to influence AMSA during CPR entering data acquired from the start of CPR and the time at which the corresponding AMSA was measured and included CPR time, average coronary perfusion pressure, number of shocks, and doses of epinephrine. We conducted several iterations examining the Akaike Information Criterion after removing the variable with the largest *P* value (ie, furthest from our critical level) and retained the model with the lowest Akaike Information Criterion value (SPSS 24.0; IBM Corp, Armonk, NY).

**Table 1:** Baseline Characteristics

	Treatment Group			
	AD (n=12)	GD (n=12)	GDAE (n=12)	*P* Value
Preparation time, min	165±32	156±24	171±51	0.614
Weight, kg	37.9±1.9	38.3±1.5	40±2.3^a^, ^b^	0.025
Core temperature, °C	37.7±0.34	37.9±0.49	37.8±0.54	0.600
Heart rate, min^−1^	118±31	118±20	125±21	0.692
Cardiac index, L/(min·m^2^)	3.76±0.77	3.56±0.60	3.75±0.70	0.802
Cardiac Stroke Work Index, cJ	26.5 (19.3–30.3)	20.8 (17.8–28.0)	22.4 (16.8–26.9)	0.551
Mean aortic pressure, mm Hg	66.8±7	65.5±12	67.4±6.7	0.872
Respiratory rate, min^−1^	29.3±3.6	32.9±4.7	33.5±5.4	0.066
P_ET_CO_2_, mm Hg	35.6±4.9	38.3±3.8	39.2±3.3	0.093
pH, aorta (unit)	7.47±0.04	7.45±0.04	7.43±0.04^a^, ^b^	0.048
Lactate, aorta, mmol/L	1.35 (1.03–1.49)	1.61 (1.12–1.82)	1.52 (1.27–1.94)	0.409
po_2_, aorta, mm Hg	222±37	214±55	199±38	0.442
pco_2_, aorta, mm Hg	37.0 (35.0–39.0)	40.0 (36.0–41.0)	42.0 (41.0–46.8)^a^, ^b^	0.006
hco_3_−, aorta, mmol/L	26.5±2.8	27.5±2.2	27.8±2.6	0.412

Baseline measurements were obtained 5 min before inducing ventricular fibrillation. Differences among groups were analyzed by 1‐way analysis of variance using the Holm–Sidak test for pairwise comparisons if the data were normally distributed; otherwise, the Kruskal–Wallis test on Ranks with Tukey test for pairwise comparisons was used, correspondingly showing means±SD or median (Q1–Q2). AD indicates AMSA‐Driven; AMSA, amplitude spectral area; GD, Guidelines‐Driven; GDAE, Guidelines‐Driven AMSA‐Enabled; P_E_ _t_co_2_, end‐tidal pco_2_.

a Different from AD.

b *P*≤0.05.

Sample sizes of 12 pigs per group were deemed adequate to detect statistically significant differences at an α level 0.05 and power 0.80 for the main effects based on previous studies by us using swine models of cardiac arrest.^37^, ^38^ For example, to detect a 120‐minute difference with a SD of 90 minutes in survival among the 3 groups, 12 animals per group would be required at an α level 0.05 and power 0.80. The data are reported in the text as proportions, mean±SD, or median with the corresponding first and third quartiles (Q1–Q3) and as specified in the Table and in figures 5 to 8. A 2‐tail *P*<0.05 was considered statistically significant.

## Results

### Baseline

There were minor but statistically significant differences in animal weight, arterial pH, and arterial Pco_2_ with GDAE different from AD after pairwise comparisons; otherwise the groups were comparable (Table).

### Shocks

A total of 123 shocks were delivered during CPR; 21 by the AD protocol (showing in Figure 1 which criteria of the AD algorithm were used); 40 by the GD protocol; and 62 by the GDAE protocol (with 2 earlier shocks delivered in 1 animal according to the *threshold criterion*).

### Survival

**Figure 3:**
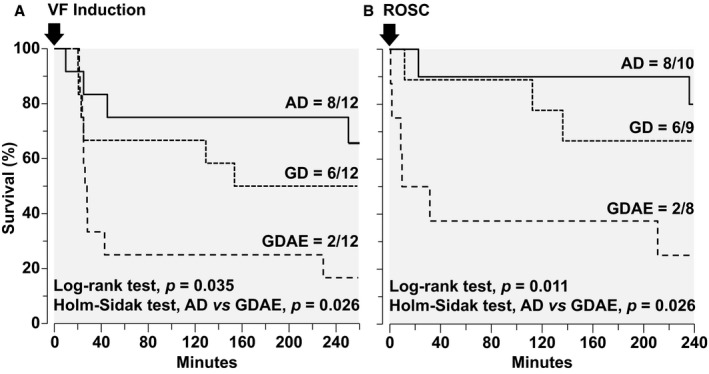
Kaplan–Meier survival curves analyzed from the time VF was induced in all 36 animals (A) and from the time ROSC was achieved in 27 animals (B), comparing the 3 defibrillation protocols: AMSA‐Driven (AD), Guidelines‐Driven (GD), and Guidelines‐Driven/AMSA‐Enabled (GDAE). The overall *P* value calculated using the log‐rank test is shown in the graph along with the pairwise comparison between AD and GDAE using the Holm–Sidak test. ROSC indicates return of spontaneous circulation; VF, ventricular fibrillation.

Kaplan–Meier curves were analyzed from the time VF was induced in all 36 animals (Figure 3A) and from the time ROSC occurred in 27 animals (Figure 3B). In both instances, survival was higher in the AD group attaining overall statistical significance and pairwise statistical significance between AD and GDAE. Likewise, the survival time from VF induction was the longest (minutes) in the AD group (198±104) followed by GD (158±109) and by GDAE (82±102), attaining overall statistical significance (*P*=0.032) and pairwise statistical significance between AD and GDAE (*P*=0.030).

We also examined whether the duration of untreated VF influenced outcome, and observed decreasing ROSC and 240‐minute survival rate with increasing duration of untreated VF but without achieving statistical significance (ie, for 6, 9, and 12 minutes of untreated VF, ROSC was 83.3%, 75.0%, and 66.7% [*P*=0.641] and for 240‐minute survival it was 58.3%, 50.0%, and 25.0% [*P*=0.232]). We also performed a Cox regression analysis of survival time assessing the effects of untreated VF duration and defibrillation protocol. Selecting the AD protocol as reference, the hazard ratios and 95% confidence intervals were 2.03 (0.57, 7.25, *P*=0.267) for GD and 4.16 (1.28, 13.50, *P*=0.018) for GDAE. Selecting the 6‐minute duration of untreated VF as reference, the hazard ratios and 95% confidence intervals were 1.06 (0.32, 3.50; *P*=0.920) for 9‐minute duration and 4.16 (1.28, 13.50, *P*=0.211) for 12‐minute duration. We also found, using 2‐way ANOVA, no statistically significant interaction between the duration of untreated VF and the defibrillation protocol on survival time (*P*=0.679).

### Interval of Untreated VF

AMSA averaged 10.1±2.3 mV·Hz 1 minute after induction of VF and 8.5±1.8 mV·Hz immediately before starting CPR (*P*≤0.001, by paired *t* test). Only minor—statistically nonsignificant—differences in AMSA were observed before starting CPR based on duration of untreated VF (ie, 8.8±1.6 mV·Hz after 6 minutes, 8.5±1.5 mV·Hz after 9 minutes, and 8.1±2.4 mV·Hz after 12 minutes, *P*=0.588). AMSA immediately before starting CPR was slightly higher in the AD group (9.0±2.4 mV·Hz) compared with GD (8.4±1.1 mV·Hz) and GDAE (7.9±1.7 mV·Hz) but without attaining statistical significance (*P*=0.338). Yet, AMSA averaged over the entire duration of untreated VF was higher in the AD group (10.4±1.6 mV·Hz) compared with GD (10.0±1.2 mV·Hz) and GDAE (8.8±1.4 mV·Hz), attaining overall statistical significance (*P*=0.027) and pairwise statistical significance between AD and GDAE (*P*=0.030).

### Resuscitation Effort—Overall Effect

**Figure 4:**
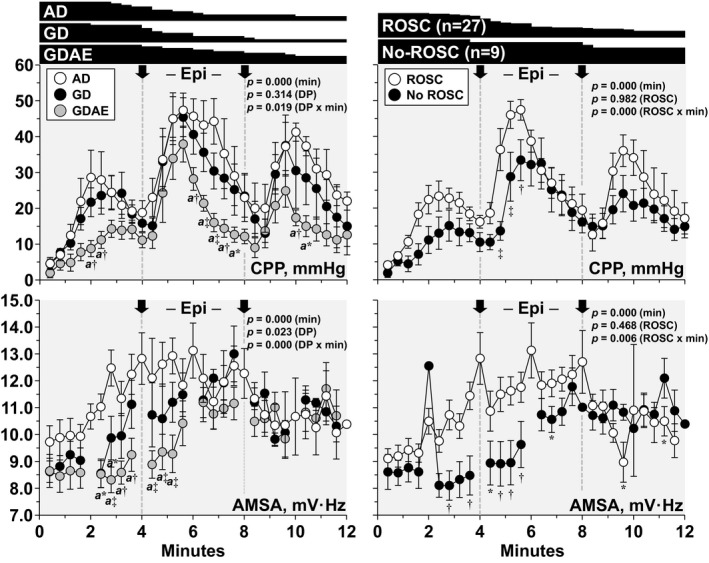
Shown are the initial 12 min of the resuscitation effort from the start of CPR (ie, min 0) depicting the main effects of the defibrillation protocols along with differences between animals that achieved ROSC and those that did not. Differences were analyzed by a linear mixed‐effect model treating time as continuous variable to assess the main effects of time (min) and either the defibrillation protocol (DP)—left—or the CPR outcome (ROSC)—right—and their interaction (DP or ROSC×min) and also treating time as a discrete variable to show for descriptive reasons differences at specific moments using the Sidak multiple comparisons pairwise method (left). AD indicates AMSA‐Driven; AMSA, amplitude spectral area; CPP, coronary perfusion pressure; CPR, cardiopulmonary resuscitation; GD, Guidelines‐Driven; GDAE, Guidelines‐Driven/AMSA‐Enabled; ROSC, return of spontaneous circulation. The number of animals receiving CPR at each time measurement is depicted graphically on top of the figure, indicating that only 10 animals remained receiving CPR after 12 min (ie, AD=3/12, GD=2/12, and GDAE=5/12). Arrows denote delivery of epinephrine (EPI) (1 mg into the right atrium) at 4 and 8 min of CPR. The absence of AMSA data in the GD and GDAE groups at 2, 4, 6, 8, 10, and 12 min corresponds to the pauses when electrical shocks were delivered, precluding calculation of AMSA. Data are shown as mean±SEM; “a” different from AD; *P≤0.05, ^†^ *P*≤0.01, and ^‡^ *P*≤0.001.

As shown in Figure 4, the coronary perfusion pressure increased gradually during the initial 2 to 3 minutes of CPR and subsequently declined until epinephrine was given, which elicited larger but also transient increases in the coronary perfusion pressure. AMSA had a different temporal profile with a gradual and largely steady increase reaching a maximum after 4 to 6 minutes. The coronary perfusion pressure and AMSA were lower in the GDAE protocol group and in animals that did not achieve ROSC. The total epinephrine given was similar in the AD group (1.2±0.9 mg) and GD group (1.0±0.9 mg) but higher in the GDAE group (1.9±1.1 mg), yet without achieving statistical significance (*P*=0.059). The number of shocks delivered was 1.5 (1.0–2.8) with the AD protocol, 2.5 (2.0–4.0) with the GD protocol, and 4.5 (3.3–8.0) with the GDAE protocol, attaining overall statistical significance (*P*=0.001) and pairwise statistical significance between AD and GDAE (*P*<0.05). The corresponding number of shocks in the 27 animals that had ROSC was 1.5 (1.0–3.0), 2.0 (2.0–3.5), and 4.0 (2.3–4.8), attaining overall statistical significance (*P*=0.037) and pairwise statistical significance between AD and GDAE (*P*<0.05). The time to ROSC (s) was 346 (258–643) in the AD group, 293 (249–430) in the GD group, and 454 (312–606) in the GDAE groups (*P*=0.308).

### First Shock Analysis

**Figure 5:**
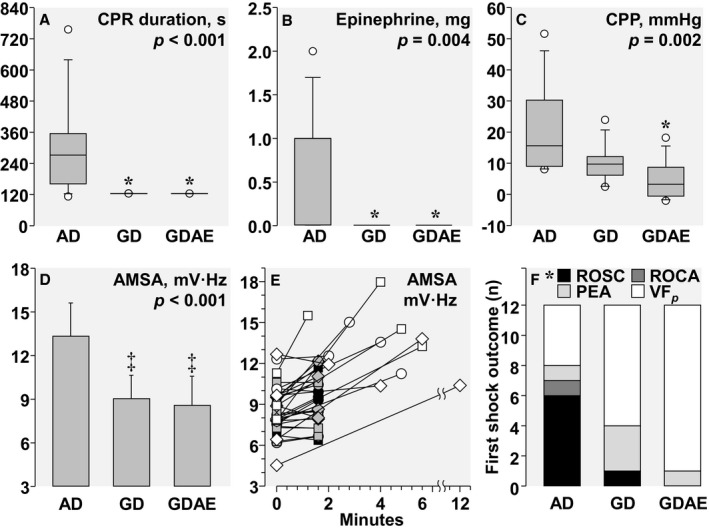
First shock analysis showing (A) CPR duration before first shock; (B) Epinephrine given before first shock; (C) Average coronary perfusion pressure (CPP) before first shock; (D) AMSA before first shock; (E) Individual AMSA values showing the AMSA immediately before start of CPR and the corresponding AMSA before shock delivery; with white symbols denoting AMSA‐Driven (AD), black symbols Guidelines‐Driven (GD), and gray symbols Guidelines‐Driven/AMSA‐Enabled (GDAE) and with the duration of untreated VF shown by circles for 6 min, by squares for 9 min, and by diamonds for 12 min (the animal that received a shock after 12 min, had a pulseless electrical activity at the end of untreated VF, and reversed to VF after 7 min if CPR, time at which the AD algorithm was activated); and (F) the first shock outcome determined at the next CPR pause or 18 s later if CPR was not resumed. The CPR duration, epinephrine, and CPP data were analyzed by the Kruskal–Wallis on ranks test and AMSA by 1‐way ANOVA with corresponding tests for pairwise comparisons. The first shock outcome was analyzed by χ^2^ (*P=0.016). The group data are shown as box plots and means±SD with the *P* values for the overall test displayed in each graph and the pairwise comparisons denoted by symbols (*P<0.05, ^‡^ *P<*0.001 vs AD). AMSA indicates amplitude spectral area; CPR, cardiopulmonary resuscitation; PEA, pulseless electrical activity; ROCA, return of cardiac activity; ROSC, return of spontaneous circulation; VF, ventricular fibrillation.

The first shock in the AD protocol was delivered after a median CPR duration of 272 s, exceeding by over 2‐fold the time to first shock in GD and GDAE groups (Figure 5A). The duration of CPR before first shock in the AD group was inversely proportional to the AMSA level immediately before start of CPR (*r*=0.866; *P*<0.001). Eight of the 12 animals in the AD group received epinephrine before the first shock but none—by protocol—in the GD and GDAE groups (Figure 5B). The longer CPR interval in the AD group before first shock was associated with higher average coronary perfusion pressure (Figure 5C) and with an ≈50% higher AMSA value (Figure 5D and 5E). The first shock success in terminating VF was 67% in the AD group, 33% in the GD group, and only 4% in the GDAE group. The corresponding rate of shocks resulting in ROSC was 50%, 8%, and 0% (*P*=0.016, Figure 5F). As CPR continued, additional animals were successfully resuscitated and at the end of the resuscitation effort, ROSC was achieved in 10 AD‐treated animals, 9 GD‐treated animals, and 8 GDAE‐treated animals (*P*=0.641).

### Epinephrine Effect in the AD Group

The 8 animals that required epinephrine—compared with the 4 that did not (because they achieved ROSC within 4 minutes)—had a lower AMSA immediately before starting CPR (7.9±1.8 vs 11.3±1.6 mV·Hz, *P*=0.010), required longer CPR duration before the first shock 299 (249–346) versus 128 (86–165) s (*P*=0.004), had a higher averaged coronary perfusion pressure but not statistically significant (28±15 vs 14±9 mm Hg, *P*=0.264) but a virtually identical AMSA before the first shock (13.1±2.5 vs 13.7±1.8 mV·Hz, *P*=0.676) and achieved comparable ROSC (7/8 vs 3/4, *P*=0.545) and 240‐minute survival (5/8 vs 3/4 *P*>0.999).

### Postresuscitation Hemodynamic Function

**Figure 6:**
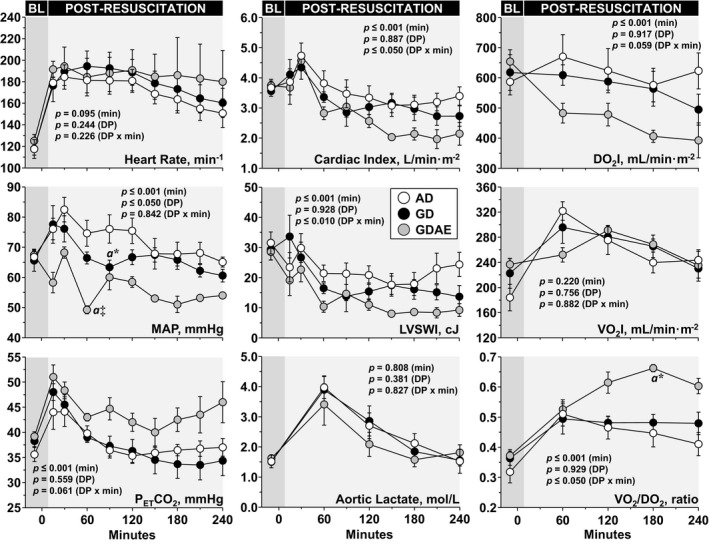
Postresuscitation effects on hemodynamic function. Baseline measurements were obtained 5 min before inducing ventricular fibrillation. Differences were analyzed by a linear mixed‐effect model treating time as continuous variable to assess the main effects of time (min) and defibrillation protocol (DP) and their interaction (DP×min) and treating each time point as a discrete moment for descriptive reasons using the Sidak multiple comparisons pairwise method to identify statistically significant differences among groups at the specific time point. AD indicates AMSA‐driven; AMSA, amplitude spectral area; DO_2_I, systemic oxygen delivery index; GD, guidelines‐driven; GDAE, guidelines‐driven/AMSA‐enabled; LVSWI, left ventricular stroke work index; MAP, mean aortic pressure; P_ET_CO_2_, end‐tidal PCO_2_; VO_2_I, systemic oxygen consumption index. The data are shown as mean±SEM; “a” different from AD*;* *P≤0.05 and ^‡^ *P*≤0.001.

Animals resuscitated with the GDAE protocol had worse hemodynamic function compared with the GD‐ and AD‐treated animals evidenced by a lower left ventricular function leading to a lower cardiac index with the highest systemic O_2_ extraction ratio (Figure 6).

### Predictive Value of AMSA

**Figure 7:**
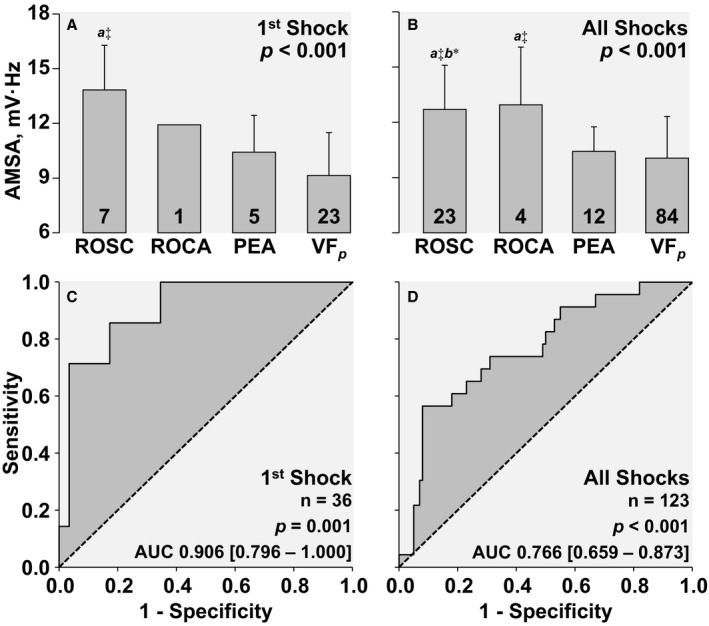
Upper graphs depict the association between AMSA values measured during the pause before shock delivery and the immediate cardiac outcomes for the first shock (A) and for all shocks (B). Differences were analyzed by 1‐way ANOVA followed by the Holm‐Sidak pairwise comparisons test with the *P* values for the overall test shown in each graph. AMSA indicates amplitude spectral area; AUC, area under the curve; PEA, pulseless electrical activity; ROCA, return of cardiac activity; ROSC, return of spontaneous circulation; VF_*p*_, persistence of ventricular fibrillation. The data are shown as mean±SD; “a” different from VF_*p*_, “b” different from PEA; *P<0.05, ^‡^ *P*<0.001. *Lower graphs* depict the areas under the receiver operator characteristic curves with their 95% confidence intervals shown in brackets for the predictive value of AMSA on the immediate outcome for the first shock outcome (C) and for all shocks (D). The outcome was defined as ROSC or no‐ROSC (ie, ROCA, PEA, or VF_*p*_).

The immediate effects of the first shock (n=36) and of all shocks (n=123)—assessed at the subsequent CPR pause (or 18 s later if CPR was not resumed)—are shown in Figure 7. The highest AMSA was observed in animals that had ROSC after the shock. Progressively lower AMSA values were associated with return of cardiac activity, pulseless electrical activity, and VF_*p*_ (Figure 7A), with the AMSA preceding ROSC higher than AMSA preceding the combined non‐ROSC outcomes (13.8±2.4 vs 9.5±2.3 mV·Hz, *P*<0.001). Likewise, analysis of all shocks showed the highest AMSA associated with ROSC and return of cardiac activity and the lowest with pulseless electrical activity and VF_*p*_ (Figure 7B), with all AMSA preceding ROSC higher than all AMSA preceding the combined non‐ROSC outcomes (12.7±2.4 vs 10.2±2.3 mV·Hz, *P*<0.001). The receiver operator characteristic curves analyzing the relationship between preshock AMSA and postshock ROSC for the first shock and for all shocks are shown in Figure 7C and 7D, demonstrating statistically significant areas under the receiver operator characteristic curves (area [95% confidence interval]) for the first shock (0.906 [0.796–1.000], *P*=0.001) and for all shocks (0.766 [0.659–0.873], *P*<0.001).

### Shock Burden

**Figure 8:**
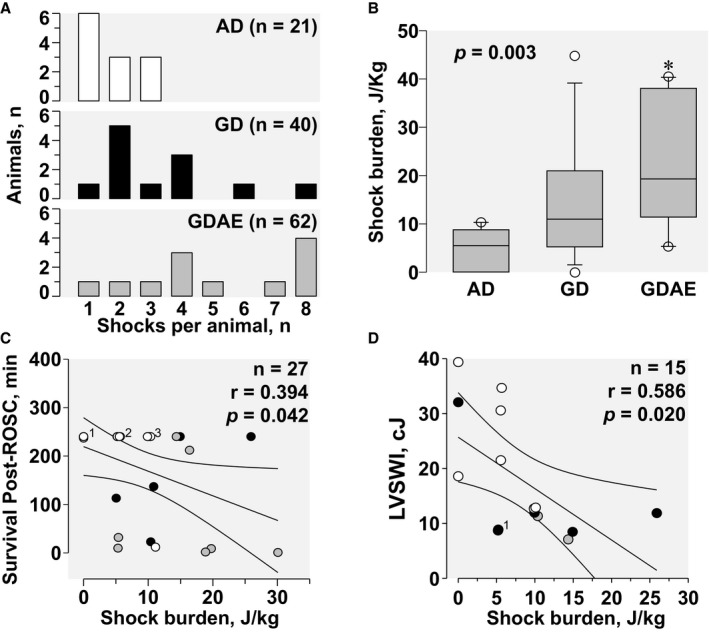
A, Histograms showing the distribution of the shocks delivered per animal according to the defibrillation protocol (AD, AMSA‐Driven; GD, Guidelines‐Driven; GDAE, Guidelines‐Driven/AMSA‐Enabled). B, Shock burden (calculated as described in the text) analyzed by Kruskal–Wallis test on ranks followed by the Tukey test for pairwise comparisons, with the data shown in box plots. The *P* value for the overall test is shown in the graph (*P<0.05 vs AD). C, Linear regression between shock burden and survival time after ROSC, showing the corresponding 95% confidence interval for the regression analysis.^1^One GD data point is behind 3 AD data points (2 at 240 min and 1 at 237 min); ^2^Two GD data points are behind 3 AD data points; ^3^One GD and 1 GDAE data points are behind the 2 AD data points. D, Linear regression between shock burden and LVSWI at 240 min in 15 animals that survived the entire postresuscitation interval, showing the corresponding 95% confidence interval for the regression analysis. Hemodynamic data were lost because of technical issues in 1 AD animal that had zero shock burden and survived 240 min. ^1^Two superimposed GD data points. AMSA indicates amplitude spectral area; LVSWI, left ventricular stroke work index; ROSC, return of spontaneous circulation.

The distribution of shocks per animal according to the defibrillation protocol is shown in Figure 8A, observing the fewer and earlier shocks given in the AD group. The shock burden—defined as the cumulative energy of shocks delivered that failed to achieve immediate ROSC—was the lowest in the AD protocol, attaining overall statistical significance (*P*=0.003) and pairwise statistical significance between AD and GDAE (*P*<0.05) (Figure 8B). In animals that attained ROSC, shock burden correlated inversely with survival time (weak correlation, *r*=0.394, *P*=0.042; Figure 8C). In animals that survived 240 minutes, shock burden correlated inversely with left ventricular stroke work index (modest correlation, *r*=0.586, *P*=0.022; Figure 8D).

### Predictors of AMSA During CPR

The linear mixed‐effect model retained CPR time (*P*=0.015), pre‐CPR AMSA (*P*<0.001), and average coronary perfusion pressure (*P*<0.001), with the following equation predictive of AMSA at a given time during CPR:

$$\begin{array}{cc} & {\text{AMSA}}_{\mathrm{ij}}\left(\text{mV}\cdot \text{Hz}\right)=2.42159+0.00094\cdot \text{CPR}\;{\text{time}}_{\mathrm{ij}}\left(s\right)+\\ & 0.69055\cdot \text{pre‐CPR}\;{\text{AMSA}}_{j}\left(\text{mV}\cdot \text{Hz}\right)+0.14909\cdot \text{average}\\ & \text{coronary perfusion}\;{\text{pressure}}_{\mathrm{ij}}\left(\text{mm Hg}\right)+e_{\mathrm{ij}}+u_{i}; \end{array}$$

where i is the value of the variable at a given time during CPR, j is the specific animal, e is the residual variance in the dependent variable, and u is the random variability around the intercept because of subject. The variance in the intercept was 0.706 mV·Hz, indicating that 95% of the AMSA values were within ±1.647 mV·Hz (ie, ±1.96√0.706) of the average AMSA at a given time during CPR.

The equation also pointed to maintaining a high coronary perfusion pressure during CPR as the most effective intervention to achieve an AMSA value associated with ROSC (ie, 13.8 mV·Hz). For example, if—on average—the pre‐CPR AMSA is 9.7 mV·Hz, a coronary perfusion pressure of 30 mm Hg would increase AMSA to 13.8 mV·Hz after 240 s, whereas a coronary perfusion pressure of 25 mm Hg would increase AMSA to only 13.1 mV·Hz after the same CPR interval.

## Discussion

The ability of AMSA to predict shock success is well documented by multiple preclinical and clinical studies,^9^, ^14^, ^15^, ^16^, ^17^, ^18^, ^19^, ^20^ and includes a recent analysis of a large clinical cohort in which an AMSA ≥15.5 mV·Hz had a positive predictive value for shock success of 84% and an AMSA ≤6.5 mV·Hz, a negative predictive value of 98%.^20^ Except for a clinical study in which a VF waveform analysis algorithm was used to decide whether to deliver a shock immediately upon VF recognition or after 2 minutes of CPR,^39^ the present study is the first—to the best of our knowledge—to investigate the use of AMSA for guiding real‐time shock delivery during CPR. The AD protocol developed by us outperformed current, time‐fixed, shock delivery protocols resulting in fewer and more precisely timed shocks associated with less postresuscitation myocardial dysfunction, leading to potentially better survival.

### AD Protocol

The central premise of our study was that AMSA provides information on the energy state of the myocardium^21^, ^22^, helping recognize myocardial readiness for successful defibrillation.^20^, ^23^, ^24^ We developed the AD algorithm to identify the optimal time for shock delivery within a 360‐s window, which is the time typically required—based on several preclinical studies—for hemodynamically effective CPR to achieve myocardial readiness after prolonged periods of untreated VF.^11^, ^25^, ^26^ It is also the time it takes AMSA to achieve a plateau.^25^, ^26^, ^28^ Yet, we acknowledge that resuscitation is a dynamic process and several variables—mainly related to the duration of untreated VF and the hemodynamic efficacy of CPR—influence myocardial readiness. We included 4 shock advisory criteria in our AD protocol intended to best recognize myocardial readiness at any time during the 360‐s time window. Of the 4 criteria, *time threshold* and *AMSA threshold* accounted for >90% of the shock advisories. This approach combined a set duration of CPR before shock delivery inversely proportional to the pre‐CPR AMSA (ie, longer CPR duration for a lower pre‐CPR AMSA) with provision for shock delivery at any time for an AMSA ≥15 mV·Hz.

The AD protocol resulted in a broad range of CPR durations before delivery of the first shock, which—on average—exceeded by >2‐fold the time to guidelines‐driven first shock and was associated with a higher averaged coronary perfusion pressure and a higher AMSA before shock delivery. The shock resulted in a higher rate of first shock success and less shock burden. These findings support the use of an AD protocol for tailoring the resuscitation effort to the individual subject in contrast to current time‐fixed shock delivery protocols tailored to population averages.

### GD Versus GDAE Protocols

The option for earlier shock delivery included in the GDAE protocol was used only twice (and in the same animal), resulting in 11 of the 12 GDAE animals receiving the same defibrillation protocol as in the 12 GD animals. Yet, the GDAE group performed worse than the GD group in several resuscitation and outcome variables and accounted for most of the statistically significant differences from the AD group after pairwise comparisons. Although block randomization proceeded uneventfully as planned, minor differences were present at baseline (Table) and AMSA averaged during the interval of untreated VF was lower in the GDAE group. The lower AMSA suggested unmeasured variables that influenced outcome. Given the relationship between AMSA and myocardial energetics, we speculate that these unmeasured variables could have acted by intensifying myocardial ischemia, reducing the responsiveness to the CPR effort.

A closer examination of the data revealed that the pre‐CPR AMSA in the GDAE group was virtually identical to the pre‐CPR AMSA in the AD subset of animals that required epinephrine (7.9±1.7 and 7.9±1.8 mV·Hz, respectively). Yet, the AD subset that required epinephrine had a survival outcome comparable to the AD subset that did not require epinephrine. At the same time, the “shock burden”—which we estimated from the cumulative number of unsuccessful shocks—was substantially lower than the shock burden in the GDAE group (19.3 [11.4–38.1] vs 5.5 [1.4–10.0], *P*=0.007), prompting us to speculate that hearts with low AMSA—indicative of a low energy state—could be more vulnerable to shock‐induced injury as discussed below.^40^, ^41^

### Shock Burden

As a result of more precise timing for shock delivery using the AD protocol, the “shock‐burden” was substantially reduced compared with the GD protocols. Current defibrillators deliver biphasic electrical shocks, which are considered safer than monophasic electrical shocks for the myocardium.^12^, ^13^ However, in a recent study—also in swine—synchronized biphasic electrical shocks delivered during spontaneous circulation (five 200‐J shocks in 5 minutes) were shown by cardiac magnetic resonance and histology to produce myocardial injury along the path of the electrical shock consistent with cell electroporation and capillary leakage leading to tissue edema.^42^ Functionally, there was reduction in left ventricular volumes indicative of diastolic dysfunction, an effect that we had previously reported after repetitive monophasic electrical shocks in an isolated rat heart model of VF.^43^ Our data suggest that shock burden could have been detrimental to the myocardium given its inverse relationship with both left ventricular function (modest association) and survival (weak association).

### Predictors of AMSA During CPR

Using a mixed linear model, we sought to identify time‐invariant and time‐variant predictors of AMSA during CPR. Seeking time‐invariant predictors, we examined variables that differed at baseline among groups, the duration of untreated VF, and the pre‐CPR AMSA (a variable that could be measured clinically). Seeking time‐variant predictors, we examine variables that could have influenced defibrillation being successful (ie, CPR time, average coronary perfusion pressure, epinephrine doses, and electrical shocks; all calculated from the start of CPR until the time of the associated AMSA measurement). Only pre‐CPR AMSA, CPR time, and average coronary perfusion pressure were statistically significant predictors of AMSA, with the largest effect associated with the pre‐CPR AMSA and the average coronary perfusion pressure. Epinephrine seems not to have had an effect independent of the effects on coronary perfusion pressure and prior shocks did not influence AMSA. The data stress the independent effect of pre‐CPR AMSA on subsequent AMSA and the critical importance of hemodynamically effective CPR, providing a rationale for exploring the use of AMSA to guide optimization of the resuscitation effort while acknowledging the influence of pre‐CPR factors.

### Predictive Value of AMSA

We confirmed the predictive value of AMSA for shock success with a very high and statistically significant area under the receiver operator characteristic curve, especially when analyzing the first shock success. Moreover, the average AMSA before a successful first shock (13.8±2.4 mV·Hz) was remarkably similar to that reported in humans by Ristagno et al^20^ (13±5 mV·Hz), although the AMSA value for unsuccessful shocks was higher in our study (9.5±2.3 mV·Hz) than in humans (6.8±3.5 mV·Hz).

### Limitations

The main limitation of our study is the healthy nature of swine used devoid of coronary artery disease, without previously compromised myocardial function, and the necessity to conduct the experiments while the animals were under anesthesia, cautioning on direct extrapolation to human settings. The minimal impact that duration on untreated VF had on AMSA and other outcome variables was intriguing and could have been in part related to the use of isoflurane anesthesia, which has been shown to exert myocardial protection during ischemia and reperfusion.^44^, ^45^, ^46^ The small outcome difference between the AD and GD groups compared with the large outcome difference between the AD and GDAE groups despite a virtually identical number of shocks delivered in the GD and GDAE groups precluded a more definitive conclusion on the superiority of the AD protocol over currently time‐fixed defibrillation protocols. In addition, our study examined the use of AMSA early in the resuscitation effort when a basic life support protocol is typically used, taking advantage of ventilation pauses to analyze AMSA without compression artifacts. A different strategy would be required if using an advanced life support protocol not necessitating ventilation pauses.

## Conclusions

We demonstrated that real‐time measurement of AMSA during CPR can be used to target the delivery of electrical shocks to the time at which there is greater probability of the shock being successful in terminating VF and reestablishing mechanically effective cardiac activity (ie, myocardial readiness for successful defibrillation). This approach reduced the delivery of ineffective and possibly damaging electrical shocks (ie, shock burden), resulting in less postresuscitation myocardial dysfunction leading to potentially better survival. Subsequent work is warranted to more precisely define the risk of shock burden and to determine whether AMSA could also be used to help guide hemodynamic optimization of the resuscitation effort before delivering an electrical shock.

## Sources of Funding

The study was supported by ZOLL Medical Corporation, Chelmsford, MA, through a research agreement established with the Resuscitation Institute at Rosalind Franklin University of Medicine and Science entitled “AMSA to Guide Shock Delivery in a Swine Model of Ventricular Fibrillation and Closed Chest Resuscitation.”

## Disclosures

Kaufman is employed by ZOLL Medical Corporation and contributed to portions of the study design, protocol implementation, and article preparation. The remaining authors have no disclosures to report.
